# An Anti-Oxidative Bioink for Cartilage Tissue Engineering Applications

**DOI:** 10.3390/jfb15020037

**Published:** 2024-02-02

**Authors:** Xin Chen, Mengni Yang, Zheng Zhou, Jingjing Sun, Xiaolin Meng, Yuting Huang, Wenxiang Zhu, Shuai Zhu, Ning He, Xiaolong Zhu, Xiaoxiao Han, Hairong Liu

**Affiliations:** 1 College of Material Science and Engineering, Hunan University, Changsha 410082, China; chenxin_hnu@hnu.edu.cn (X.C.); yangmengni@hnu.edu.cn (M.Y.); xlinmeng@163.com (X.M.); 13487573006@163.com (Y.H.); wenxiang@hnu.edu.cn (W.Z.); shuai031318@hnu.edu.cn (S.Z.); 2 College of Biology, Hunan University, Changsha 410082, China; jingjingsun@hnu.edu.cn; 3 College of Mechanical and Vehicle Engineering, Hunan University, Changsha 410082, China

**Keywords:** antioxidant, bioink, 3D bioprinting, rutin, cartilage tissue engineering

## Abstract

Since chondrocytes are highly vulnerable to oxidative stress, an anti-oxidative bioink combined with 3D bioprinting may facilitate its applications in cartilage tissue engineering. We developed an anti-oxidative bioink with methacrylate-modified rutin (RTMA) as an additional bioactive component and glycidyl methacrylate silk fibroin as a biomaterial component. Bioink containing 0% RTMA was used as the control sample. Compared with hydrogel samples produced with the control bioink, solidified anti-oxidative bioinks displayed a similar porous microstructure, which is suitable for cell adhesion and migration, and the transportation of nutrients and wastes. Among photo-cured samples prepared with anti-oxidative bioinks and the control bioink, the sample containing 1 mg/mL of RTMA (RTMA-1) showed good degradation, promising mechanical properties, and the best cytocompatibility, and it was selected for further investigation. Based on the results of 3D bioprinting tests, the RTMA-1 bioink exhibited good printability and high shape fidelity. The results demonstrated that RTMA-1 reduced intracellular oxidative stress in encapsulated chondrocytes under H_2_O_2_ stimulation, which results from upregulation of *COLII* and *AGG* and downregulation of *MMP13* and *MMP1*. By using in vitro and in vivo tests, our data suggest that the RTMA-1 bioink significantly enhanced the regeneration and maturation of cartilage tissue compared to the control bioink, indicating that this anti-oxidative bioink can be used for 3D bioprinting and cartilage tissue engineering applications in the future.

## 1. Introduction

In recent years, 3D bioprinting has emerged as a powerful biofabrication technology with a wide range of applications in various fields such as tissue engineering, regenerative medicine, personalized medicine, drug discovery, and assay research [1,2]. Among them, light-cured 3D bioprinting is known for its high precision, fast curing, and gentle handling of biomaterials, which has led to its widespread use in cartilage tissue engineering. Many light-cured bioinks based on natural/synthetic polymers, such as glycidyl methacrylate-modified silk protein (SFGMA) [3], glycidyl methacrylate-modified hyaluronic acid [4], methacrylate-modified gelatin [5,6], and methacrylate-modified collagen [7], have been used for cartilage bioprinting. Multifunctional bioactive inks are the key to adapting 3D bioprinting technology to different application situations [8,9]. Current research predominantly assesses hydrogel composition and physical properties, such as matrix stiffness, in supporting chondrocyte phenotypes and influencing in vitro cartilage-specific extracellular matrix (ECM) generation [10,11]. However, the impact of oxidative stress and inflammatory microenvironments in osteoarthritis (OA) cartilage on the reparative potential of bioprinted constructs using current bioinks has been neglected [12].

Reactive oxygen species (ROS) are important for chondrocyte function. Excess ROS can disrupt the cellular phenotype by disrupting the balance between anabolic and catabolic metabolism of the ECM components, affecting the normal function of chondrocytes, leading to progressive destruction of cartilage integrity and preventing self-repair of cartilage tissues; in addition, ROS can synergize with many pro-inflammatory cytokines to promote OA inflammation [13,14,15]. Many antioxidants (delivered as free molecules or encapsulated in nanoparticles or microparticles) have been introduced to reduce excess ROS; however, the instability, rapid diffusion, and biocompatibility of these small-molecule antioxidants at the target site have hampered the prospects of their application [16,17,18]. For free molecules, the release of antioxidant small molecules from bioinks is difficult to control because of the potential for sudden release [19]. In order to achieve long-term release, an antioxidant chemical is grafted to high molecular weight biomaterials, which can gradually release antioxidant molecules with the degradation of this biomaterial by living cells [16,17]. Therefore, an antioxidant delivery strategy with high reactive oxygen species scavenging performance is urgently needed, such as the construction of a bioink with natural antioxidant properties that can support the growth and function of chondrocytes while safeguarding them from changes and damage induced by oxidative stress and inflammation.

Rutin (quercetin-3-o-rutinoside) is a small bioactive molecule purified from plants and is known for its potent anti-inflammatory and antioxidant properties [20,21]. In addition, its osteochondroprotective effects have been reported in cellular and/or animal models, promoting chondrocyte proliferation and cartilage phenotype maintenance and alleviating cartilage extracellular matrix degradation [22,23,24]. It has been shown that rutin inhibits age-induced inflammatory response and extracellular matrix (ECM) degradation by targeting NF-κB/MAPK pathway proteins BCL-2 and TRAF-6 [25]. It has been shown that rutin effectively reduced the expression levels of inducible nitric oxide synthase *(iNOS)*, cyclooxygenase-2 *(COX-2)*, tumor necrosis factor-α *(TNF-α)*, and matrix metalloproteinase 13 (*MMP-13*), and increased the expression of *COL II* and aggrecan [26]. Therefore, rutin has been modified as an anti-oxidative component, which is photo-curable, to compose the bioink with natural antioxidant properties, with an additional component that constructs the frame of hydrogel following solidification like SFGMA.

**Figure 1:**
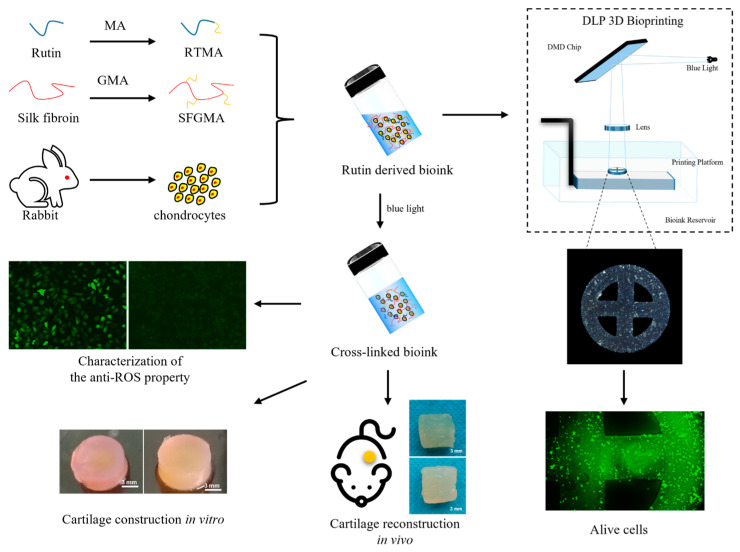
Anti-oxidative bioink for cartilage tissue engineering application.

A variant would be based on the logical scheme presented in Figure 1. We prepared an anti-oxidative bioink using photo-crosslinkable methacrylate-modified rutin as the antioxidant component and methacrylate silk fibroin as the biomaterial component. The microstructure, degradation, and free radical scavenging properties of the bioink hydrogels with different compositions were investigated. The question of whether this anti-oxidative bioink reduces the oxidative stress in chondrocytes was examined and the printability of this anti-oxidative bioink was tested using a projected 3D bioprinter. Finally, in vitro and in vivo experiments were employed to assess whether this anti-oxidative bioink promotes cartilage regeneration.

## 2. Materials and Methods

### 2.1. Materials

Rutin, glycidyl methacrylate (GMA, 94%), and methacrylate anhydride (MA, 94%) were purchased from Shanghai Aladdin Biochemical Technology Co., Ltd. (Shanghai, China). Silkworm cocoon was purchased from Shenzhen Mermaid Artistry Makeup Co., Ltd. (Shenzhen, China). BALB/c mice (male, 6–8 weeks old) were purchased from Hunan Slake Jingda Experimental Animal Co., Ltd. (Changsha, China).

### 2.2. Synthesis of RTMA

**Figure 2:**
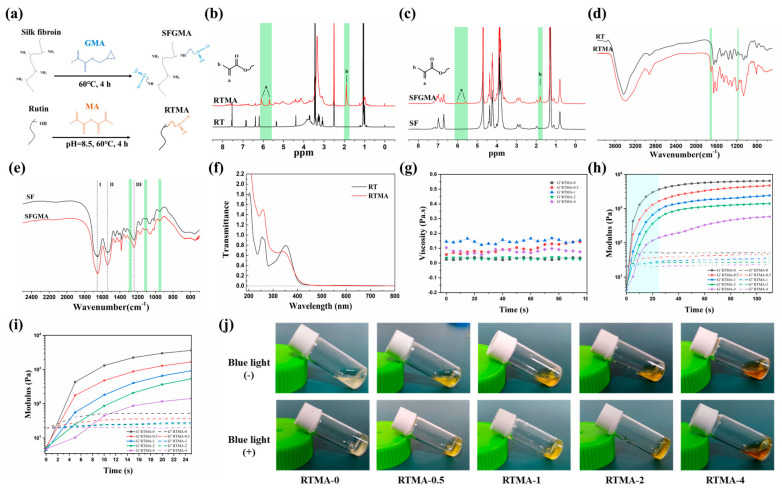
Synthesis and characterization of RTMA and SFGMA. (**a**) Synthesis diagram of RTMA and SFGMA. (**b**) 1H NMR spectra of rutin and RTMA in DMSO-d6. a: double-bond protons, b: methyl protons. (**c**) 1H NMR spectra of SF and SFGMA in D_2_O. a: double-bond protons, b: methyl protons. (**d**) FTIR spectra of rutin and RTMA. (**e**) FTIR spectra of SF and SFGMA. I, II and III: peaks of amide. (**f**) UV-vis spectra of rutin and RTMA. (**g**) Viscosity characterization and (**h**,**i**) dynamic time-sweep rheological analysis of anti-oxidative bioink candidates. (**j**) Gelation of anti-oxidative bioink candidates before (up) and after (down) exposure to blue light.

For methacrylation, 2.0 g of rutin was dissolved in 200 mL of PBS solution and stirred for 2 h at 60 °C. The solution was then combined with 1 mL of MA and stirred for 4 h at 60 °C in the dark. To maintain a pH between 8.0 and 8.5 throughout the modification reaction, 1 M NaOH solution was added as needed. The resulting solution was neutralized with 0.1 M HCl before undergoing a 5-day dialysis against distilled water. The dialyzed reaction solution was lyophilized to obtain the RTMA, which was stored at 4 °C for further analysis. Figure 2a provides a schematic representation of the methacrylation process involving rutin and MA.

### 2.3. Synthesis of SFGMA

SFGMA was synthesized following the methodology outlined in references [27,28]. Briefly, 50 g of sliced cocoons were cooked for 30 min at 100 °C in 1 L of 0.05 M Na_2_CO_3_ solution to remove sericin. The degummed SF obtained was dried for 48 h. To prepare SFGMA (depicted in Figure 2a), 10 g of degummed SF was dissolved in a 9.3 M lithium bromide solution at 60 °C for 2 h. Subsequently, 3 mL of GMA was added, and the mixture was stirred for 4 h at 60 °C to facilitate the reaction between SF and GMA. The resulting solution underwent 7 days of dialysis with distilled water, followed by lyophilization. The lyophilized product was stored at −80 °C for future use.

### 2.4. Characterization of RTMA and SFGMA

RTMA and SFGMA underwent analysis using FTIR (Thermo Nicolet Nexus 470, Waltham, MA, USA), UV-vis (UV-2600, Shimadzu Corporation, Kyoto, Japan), and 1H NMR (Bruker 400 MHz Advance, Ferranden, Switzerland).

### 2.5. Preparation of Hydrogels

For the preparation of the bioink precursor solution, RTMA, SFGMA (15 wt%), and the photo initiator LAP (0.5 wt%) were dissolved in PBS solution (pH 7.4). The concentration of RTMA was varied to 0, 0.5, 1, 2, and 4 mg/mL. The resulting combinations were exposed to blue light (405 nm) to generate photo-crosslinkable hydrogels, denoted as RTMA-0, RTMA-0.5, RTMA-1, RTMA-2, and RTMA-4, respectively.

### 2.6. Characterization of Hydrogels

The microscopic morphology of lyophilized photo-crosslinked hydrogel samples prepared as established in Section 2.5 were examined following the protocol outlined in a previous publication [29]. The swelling behavior, the compression properties, and the collagenase degradation of photo-crosslinked hydrogel samples were investigated following the protocol outlined in our previous publication [30,31].

### 2.7. Rabbit Chondrocyte Isolation, Culturing, and Hydrogen Peroxide (H_2_O_2_) Treatment

All animal experiments in this study received approval from the Animal Care and Ethics Committee of the College of Biology, Hunan University. The isolation and culturing of rabbit chondrocytes were conducted following the protocol outlined in a previous publication [30].

To prepare chondrocyte-laden bioink, in total, 2 × 10^6^ chondrocytes were initially suspended in 1 mL of precursor solution, prepared in accordance with the method outlined in Section 2.5. Subsequently, each 100 µL of the bioink was light-cured and incubated in culture medium for subsequent experiments.

In this study, the chondrocyte-laden hydrogel samples were treated with H_2_O_2_ to simulate oxidative stress, following the protocol outlined in reference [32]. In summary, each piece of cell-laden hydrogel sample was placed into a separate well of a 24-well plate, and an additional 1 mL of DMEM containing 1 mM H_2_O_2_ was added to each well. The samples were then cultured for an additional 24 h before harvesting.

### 2.8. Cell Proliferation

Chondrocyte-laden hydrogel samples were formed by photo-crosslinking the bioink precursor solution, prepared as outlined in Section 2.5, with added chondrocytes. Cell proliferation in these samples was assessed by using fluorescein diacetate (FDA) staining and alamarBlue assays, following the protocol established in our previous publication [29].

### 2.9. 3D Bioprinting

The bioink precursor solution, containing chondrocytes at a density of 2 × 10^6^ cells/mL, was 3D printed into designed shapes using a DLP printer (nanoArch S140, BMF Material Technology Inc., Shenzhen, China). Briefly, the bioink was photocured at an exposure intensity of 22 mW/cm^2^ and 14 mW/cm^2^ with an exposure time of 10 s and 8 s per 100 μm, and the 3D bioprinting parameters are shown in Table 1. The printed samples were examined using a digital microscope (Dino-Lite, ANMO ELECTRONICS Corporation, Wuxi, China). After 7 days of culture, live cells in the test samples were observed by using an inverted fluorescence microscope (IX-73, Olympus, Tokyo Metropolis, Japan) following FDA staining.

### 2.10. In Vitro Antioxidant Property Evaluation

The antioxidant capacity of the samples was assessed by scavenging 1,1-diphenyl-2-picrylhydrazyl (DPPH) free radicals, following the methodology outlined in the previously published article [29]. In summary, 1 g of the photo-crosslinked hydrogel sample was dispersed in 2 mL of ethanol containing 200 μM DPPH, stirred in the dark for 30 min, and subsequently analyzed using UV–visible spectroscopy.

### 2.11. ROS Responsive Fluorescent Staining

A H_2_O_2_-responsive fluorescent probe (H2DCFDA, Millipore Corp., Shanghai, China) was utilized to examine the in vitro H_2_O_2_ scavenging effect of hydrogel samples on cells, following the protocol outlined in reference [32]. Briefly, chondrocytes were first equilibrated with DMEM for 1 h, followed by incubation for an additional 1 h at 37 °C with the leachate obtained by soaking each hydrogel sample in DMEM culture solution containing 500 µM of H_2_O_2_ for 1 h. Then, the cells were further incubated followed by incubation with a 5 µM probe solution for 30 min. After removing the probe solution, microtiter plate readings and microscopic imaging were conducted.

### 2.12. Cartilage Regeneration Evaluation In Vitro and In Vivo

To assess in vitro cartilage regeneration, 4 × 10^6^ chondrocytes were seeded onto photo-crosslinkable hydrogels prepared as described in Section 2.5, forming cell–hydrogel constructs. After 2 weeks of in vitro incubation, the transcriptional levels of chondrogenic genes were evaluated using RT-qPCR assays.

For in vivo cartilage regeneration assessment, hydrogel samples containing encapsulated chondrocytes (2 × 10^6^ cells/mL) were subcutaneously implanted into BALB/c mice. After 28 days, all samples were retrieved and histological analysis of each sample was conducted to evaluate the efficiency of in vivo cartilage regeneration.

### 2.13. RNA Isolation and RT-qPCR Analysis

The isolation of total RNA and the real-time quantitative polymerase chain reaction (RT-qPCR) assay were performed according to our previous publication [29]. The primers (Table 2) of the test genes for RT-qPCR were designed by using the oligo 7.0 software and their sequence was validated by BLAST on the NCBI website.

### 2.14. Statistical Analysis

All data in this research are expressed as the mean ± standard deviation *n* ≥ 3. Significant differences between the groups were determined with the help of the GraphPad Prism 9.0 program. The differences were considered significant at *p* < 0.05 (*), *p* < 0.01 (**), *p* < 0.001 (***), and *p* < 0.0001 (****).

## 3. Results and Discussion

### 3.1. Synthesis and Characterization of RTMA and SFGMA

RTMA, synthesized through MA grafting onto rutin, served as the bioactive component, while SFGMA, obtained by reacting with GMA, acted as the biomaterial component of the bioink. Successful preparation of RTMA and SFGMA was confirmed by 1H NMR, FTIR, and UV-vis, following the protocol outlined in Section 2.2 (Figure 2a). The 1H NMR spectra validated the successful conjugation of methacryloyl groups onto rutin and SF, with new peaks at δ 5.7 ppm and 6.1 ppm for double-bond protons and δ 1.9 ppm for methyl protons (Figure 2b,c). Additionally, FTIR spectroscopy of RTMA confirmed rutin methacrylation, evidenced by new peaks at 1710 cm^−1^ and 1172 cm^−1^ corresponding to C=O and -C-O-C- stretching vibration peaks, respectively (Figure 2d). The FTIR spectra (Figure 2e) of SFGMA exhibited characteristics consistent with a referenced article [33]. These data illustrate the successful manufacturing of RTMA and SFGMA for further investigation. The grafting of MA onto rutin induced a significant change in the characteristic UV absorption peak of RTMA compared to rutin (Figure 2f). The light absorption of RTMA at 400–450 nm confirms its wavelength range, suggesting its ability to absorb scattered light during the light-curing printing process based on 405 nm irradiation. In addition, Figure 2g shows that the viscosity of the RTMA bioinks remained relatively stable over time and had a viscosity suitable for light-cured bio-3D printing. Dynamic time-scanning rheological analysis (Figure 2h) shows that under 405 nm laser irradiation, the RTMA prepolymer solution achieved light curing in about 30 s, with RTMA-4 failing to fully gel after more than 60 s. Figure 2i, a zoomed-in version of Figure 2h, also shows that the onset of gelation of RTMA bioinks becomes longer as the proportion of RTMA increases. The above shows that RTMA-0, RTMA-0.5, RTMA-1, and RTMA-2 have good light-curing rheological properties and can be used for light-curing bio-3D printing. Gel-forming experiments, conducted according to the protocol in Section 2.5, demonstrated that RTMA-0, RTMA-0.5, RTMA-1, and RTMA-2 solutions containing 0, 0.5, 1, and 2 mg/mL of RTMA, respectively, solidified after 60 s of exposure to blue light (405 nm) (Figure 2j). However, the RTMA-4 solution with 4 mg/mL of RTMA did not undergo photo-crosslinking, consistent with its higher blue light absorption capacity (Figure 2f). These results confirm the successful synthesis of RTMA and SFGMA for further investigation.

### 3.2. Characterization of the Bioink Precursor Solutions and Prepared Hydrogels

**Figure 3:**
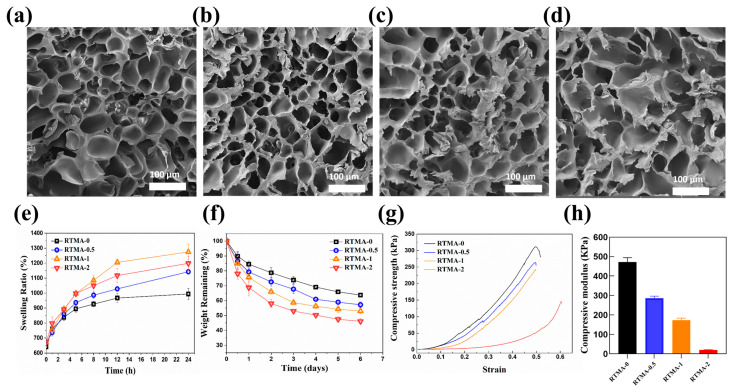
Characterization of anti-oxidative bioink candidates. (**a**–**d**) Micro-morphology of lyophilized RTMA-0, RTMA-0.5, RTMA-1, and RTMA-2. (**e**) Swelling ratio of RTMA-0, RTMA-0.5, RTMA-1, and RTMA-2. (**f**) In vitro collagenase degradation of RTMA-0, RTMA-0.5, RTMA-1, and RTMA-2. (**g**) Compressive stress–strain curve and (**h**) compressive modulus of RTMA-0, RTMA-0.5, RTMA-1, and RTMA-2.

Bioink precursor solutions with concentrations of 0.5, 1, and 2 mg/mL of RTMA, along with SFGMA, were prepared and photocured following the protocol outlined in Section 2.4. The sample containing 0% RTMA was used as a control. The microstructure of lyophilized samples of solidified bioink precursor solutions (Figure 3a–d) were characterized by SEM. The microstructures revealed interconnected porous formations, with average pore sizes ranging from 55 to 125 μm, as determined by ImageJ analysis of SEM images. These results indicated that the porous structures are conducive to cell adhesion, migration, and nutrient/metabolic waste transport. In vitro swelling behavior and collagenase degradation of the anti-oxidative bioink bioinks were influenced by RTMA concentration (Figure 3e,f). The equilibrium swelling rates exceeded 900%, indicating that they are suited to store nutrients effectively in the moist environment of synovial joints. After 6 days of collagenase incubation, degradation ratios for RTMA-0, RTMA-0.5, RTMA-1, and RTMA-2 were 36.29 ± 1.38%, 42.81 ± 1.84%, 47.05 ± 1.89%, and 53.91 ± 1.73%, respectively (Figure 3f). The compressive strength of RTMA-0 was 310 kPa, while the other groups exhibited a reduction, ranging from 250 to 100 kPa, attributed to RTMA incorporation (Figure 3g). The ideal scaffold for cartilage tissue engineering should mimic the Young’s modulus (10 MPa) of cartilage; however, the mechanical properties of natural photo-crosslinked hydrogels are poor [34]. For example, the compression modulus of GelMA and SerMA is only 5–30 kPa and 10–40 kPa. In contrast, SilMA has very strong mechanical properties with a compression modulus of about 390–910 kPa, which is 30 times higher than that of GelMA. In this study, the compressive modulus of RTMA-0 was 472 kPa, while the other groups exhibited a reduction, ranging from 287 to 19 kPa, attributed to RTMA incorporation (Figure 3h). Although it is still far from the ideal Young’s modulus, it is slightly better than the mechanical properties of commonly used natural photo-crosslinked hydrogels. These findings confirm that the photo-curability of all bioink precursor solutions, with their solidified hydrogels, are suitable for cell adhesion, migration, and nutrient/waste transportation.

### 3.3. Biocompatibility and Proliferation Testing In Vitro

**Figure 4:**
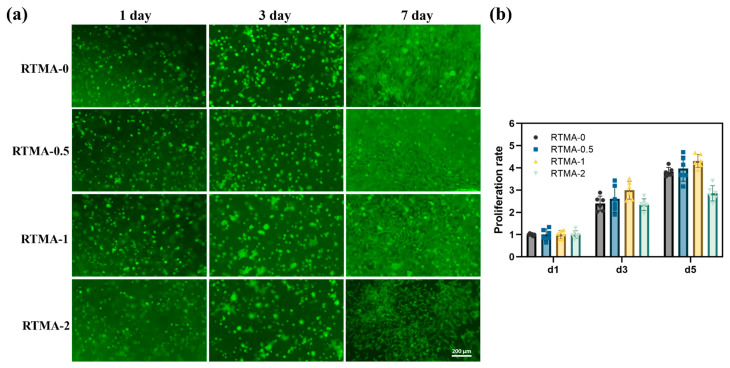
Cell culture with anti-oxidative bioink candidates. (**a**) Photographs of living cells wrapped in bioink candidates from 1 to 7 days. (**b**) Proliferation rate of chondrocytes in bioink candidates.

Before utilizing cross-linked bioinks for in vivo tissue engineering applications, evaluating their biocompatibility in vitro is essential [35]. The biocompatibility of hydrogel samples was assessed using chondrocytes, which were encapsulated in RTMA-0, RTMA-0.5, RTMA-1, and RTMA-2. The samples were then incubated with cell culture media for 1, 3, and 7 days, and the proliferation of chondrocytes in the hydrogel samples was qualitatively determined using FDA staining. As depicted in Figure 4, encapsulated chondrocytes in RTMA-0, RTMA-0.5, and RTMA-1 proliferated after seven days of incubation, exhibiting good cell adhesion properties. Among them, RTMA-1 demonstrated the highest cell density, suggesting superior biocompatibility compared to the other samples. Furthermore, the proliferation of encapsulated chondrocytes was quantified using the alamarBlue assay (Figure 4b), providing additional confirmation of the previously observed results. Hence, RTMA-1 exhibited favorable biocompatibility for tissue engineering applications and was selected for further testing, with RTMA-0 as the control.

### 3.4. Testing the Bioink for 3D Bioprinting Applications

**Figure 5:**
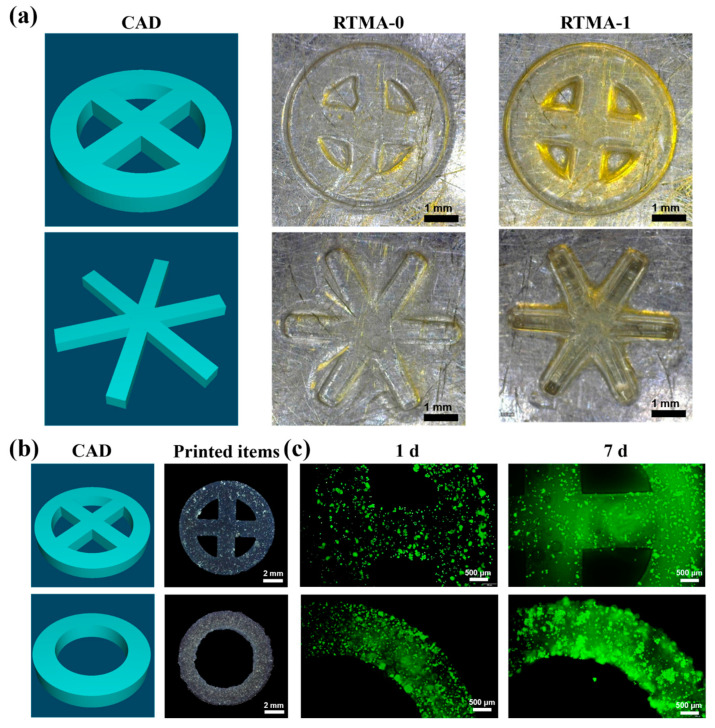
(**a**) The 3D digital model and the printed constructs of the RTMA-0 and RTMA-1. (**b**) Top view of the cell-loaded RTMA-1 printed constructs and (**c**) FDA staining after 7 days of culture.

Considering the biocompatibility and stability, RTMA-1 was selected for bio-3D printing tests, and the structural fidelity of its printed products was evaluated, with RTMA-0 as the control. The results show that the structures of hydrogel products printed with the RTMA-1 displayed a higher shape fidelity than the control bioink (Figure 5a). The light absorption of RTMA (Figure 2f) around wavelengths from 400 nm to 450 nm, which corresponds with conjugated large π-bonds and double bonds of its molecule, limited the scattering of light at 405 nm to enhance the fidelity of 3D bioprinting [36]. Compared with the control bioink, RTMA-1 enhanced the fidelity of 3D printing, which allowed printed items to more closely resemble their designed and required shape.

The distinct refractive index of cells compared to bioinks leads to light scattering during the bioprinting process, posing challenges to printing [8]. To further evaluate the printing performance of cell-loaded bioinks, additional 3D printing tests were conducted. Following bioprinting, the outlines of the printed hydrogel products maintained the designed digital shapes with high fidelity, highlighting the potential of RTMA-1 as a bioink with excellent print fidelity for 3D bioprinting (Figure 5b). The printed hydrogel products underwent FDA staining after 7 days of in vitro culture to qualitatively assess cell proliferation and activity in the test samples. As depicted in Figure 5c, chondrocytes were uniformly distributed in all printed samples, and cell proliferation was observed. This suggests that RTMA-1 holds promise for tissue engineering applications, particularly in cartilage tissue engineering.

### 3.5. Anti-Oxidative Property of the Bioink

**Figure 6:**
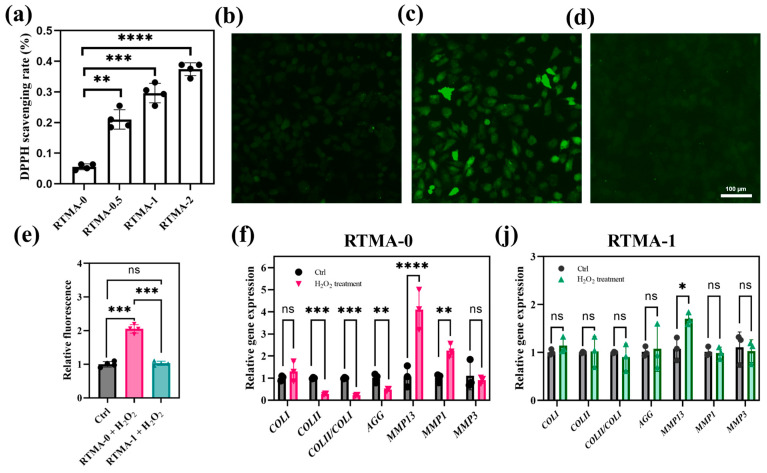
(**a**) Antioxidant property of bioink hydrogel (n = 4). (**b**–**e**) ROS fluorescent staining of chondrocytes (**b**) without H_2_O_2_ exposure, (**c**) with H_2_O_2_ and RTMA-0, (**d**) with H_2_O_2_ and RTMA-1. (**e**) Quantitative measurement of the intracellular ROS levels above (n = 4). Effects of excess H_2_O_2_ on the regulation of cartilage extracellular matrix-related genes and catabolic genes after chondrocytes were exposed to H_2_O_2_ when cultured in (**f**) RTMA-0 and (**j**) RTMA-1 (n = 3). Significant differences symbols: ns = no significant difference; * = *p* < 0.05; ** = *p* < 0.01; *** = *p* < 0.001 and **** = *p* < 0.0001.

The bioink hydrogel samples, featuring varied RTMA contents, underwent comparison of their free radical scavenging ability (Figure 6a) through the DPPH assay. Notably, RTMA-0 hydrogels displayed modest antioxidant activity, likely attributed to the intrinsic properties of the hydrogen donor -OH group. The antioxidant efficacy experienced a pronounced boost with escalating RTMA content in the samples. The observed escalation in free radical inhibitory activity, progressing from 5.6% in the RTMA-0 hydrogel to 21.0% in the RTMA-0.5 hydrogel, 29.6% in the RTMA-1 hydrogel, and reaching 37.4% in the RTMA-2 hydrogel, signifies a proportional augmentation in the presence of phenolic derivatives. This increase contributes to the improved hydrogen-donating capacity of the bioink, thereby exhibiting notable in vitro antioxidant activity.

To assess the potential of hydrogels in alleviating cellular oxidative stress, chondrocytes were cultured in vitro with the leachate (denoted as the RTMA-0 or RTMA-1 group) obtained by soaking RTMA-0 or RTMA-1 hydrogel samples in DMEM culture with H_2_O_2_ for 2 h. Intracellular ROS levels were assessed using the H_2_O_2_-responsive probe H2DCFDA, which is converted to a fluorescent product upon activation by intracellular H_2_O_2_ [37].

As depicted in Figure 6b–d, the percentage of fluorescent cells in the RTMA-0 group was markedly higher than that in the control and RTMA-1 groups. Similarly, the fluorescence intensity per well was significantly elevated in the RTMA-0 group compared to the control and RTMA-1 groups. Notably, there was no substantial difference in the average fluorescence intensity between the control and RTMA-1 groups (Figure 6e), indicating that RTMA-1 hydrogel effectively scavenges H_2_O_2_ from the culture medium, mitigating oxidative stress in cultured cells in vitro.

To further assess the biological effects and antioxidant properties of hydrogels on chondrocytes, we examined the impact of excess H_2_O_2_ on the regulation of cartilage extracellular matrix-related genes (*AGG*, *COL I*, and *COL II*) and catabolic genes (*MMP13*, *MMP1*, *MMP3*) through RT-PCR (Figure 6f–j). Chondrocytes encapsulated in RTMA-0 hydrogels without H_2_O_2_ scavenging capacity exhibited significant decreases in *COL II*, *COL II/COL I,* and *AGG* transcripts, along with significant upregulation of *MMP13* and *MMP1* after exposure to H_2_O_2_. It recapitulates the transcriptional changes in chondrocytes under oxidative stress during the development of osteoarthritis [38]. In contrast, chondrocytes in RTMA-1 bioink maintained similar transcript levels in the presence of H_2_O_2_, with *MMP13*, although not fully maintained, exhibiting reduced differences compared to controls. The H_2_O_2_ scavenging ability exhibited by RTMA-1 bioink is derived from RTMA-1, a naturally occurring flavonoid rutin methacrylation derivative. RTMA-1 possesses excellent water solubility compared to rutin, along with preserved antioxidant properties, and is capable of covalently binding with the components of bioink material, thereby endowing it with antioxidant functionality. In this context, RTMA-1, in conjunction with RTMA-0 and live chondrocytes, constitutes an anti-oxidative bioink capable of significantly reducing intracellular oxidative stress and maintaining transcript levels of chondrocyte function-related genes in loaded chondrocytes.

### 3.6. Cartilage Regeneration Evaluation In Vitro and In Vivo

**Figure 7:**
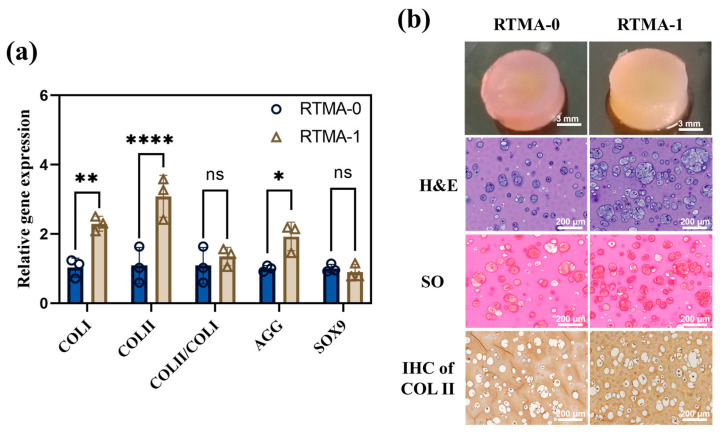
(**a**) The transcript levels of chondrogenesis-related genes in chondrocytes encapsulated in RTMA-0 and RTMA-1 hydrogels after 7 days of in situ culture. (**b**) Overall view and histological staining, i.e., H&E, Safranin-O, and Collagen II staining, of cell-loaded RTMA-0 and RTMA-1 samples cultured for 4 weeks in vitro. Significant differences symbols: ns = no significant difference; * = *p* < 0.05; ** = *p* < 0.01 and **** = *p* < 0.0001.

We assessed the transcript levels of chondrogenesis-related genes *COL I*, *COL II*, *AGG*, and *SOX9* in chondrocytes by using RT-qPCR to explore the influence of RTMA-1 on gene regulation in encapsulated chondrocytes. In comparison to chondrocytes in the control samples, RTMA-1 significantly enhanced the transcription of *COL II* and *AGG* in chondrocytes (Figure 7a). Given that *COL II* and proteoglycan are pivotal components of the cartilage extracellular matrix, these findings suggest that RTMA-1 may stimulate cartilage-specific extracellular matrix synthesis, leading to improved cartilage tissue regeneration. The presence of RTMA in this bioink appears to significantly enhance cartilage tissue regeneration by upregulating chondrogenesis-related genes.

To assess the efficacy of RTMA-1 bioink in tissue engineering applications, solidified hydrogel samples from RTMA-1 bioink and the control bioink, both carrying chondrocytes at a density of 4 × 10^6^ cells/mL, were cultured in cell culture medium for 28 days. The samples were then sectioned and analyzed using H&E staining, Safranin O (SO) staining, and COL II staining to evaluate cartilage-like tissue regeneration efficiency in vitro. H&E images (Figure 7b) revealed that RTMA-1 bioink promoted the formation of cartilage luminal space and enhanced cartilage-like tissue regeneration, as indicated by SO and COL II staining, compared to the control sample. In addition, the above studies found that the innate antioxidant properties acquired by RTMA-1 bioinks do not negatively affect the chondrocyte-related functions.

**Figure 8:**
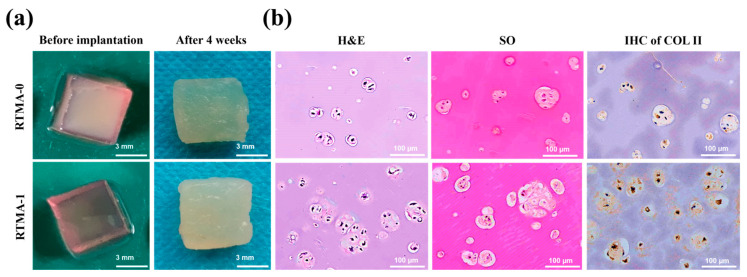
Subcutaneous implantation in vivo. (**a**) Overall view of the sample before subcutaneous implantation and after subcutaneous implantation in BALB/c mice for 4 weeks. (**b**) Histological staining, i.e., H&E, Safranin-O, and Collagen II staining, of samples implanted subcutaneously in BALB/c mice for 4 weeks.

Evaluation of in vivo tissue regeneration is crucial for determining the suitability of biomaterials in tissue engineering [39]. To further evaluate the RTMA-1 bioink for tissue engineering applications, photo-crosslinked hydrogel samples containing chondrocytes were subcutaneously implanted into BALB/c mice for 4 weeks. Compared to specimens prepared with the control bioink, the RTMA-1 bioink significantly enhanced cartilage lumen formation and deposition of cartilage-specific ECM (Figure 8b), demonstrating its promotion of cartilage tissue regeneration in vivo. Additionally, after 4 weeks of implantation, the appearance and color of the RTMA-1 bioink specimens closely resembled natural cartilage tissue (Figure 8a). Although future animal models of cartilage defects are needed to fully characterize their tissue repair effects, these findings may still underscore the potential of RTMA-1 bioink in cartilage tissue regeneration, as well as for use as a bioink combination strategy to enhance cartilage defect repair in environments with oxidative stimuli.

## 4. Conclusions

In summary, we developed an anti-oxidative bioink with RTMA as an additional bioactive component and SFGMA as a biomaterial component. This bioink is cytocompatible, 3D bioprintable, and exhibits antioxidant properties. Our findings indicate that RTMA-1 bioink enables precise 3D bioprinting of hydrogel products with high fidelity and promotes cell proliferation in printed samples. Moreover, the bioink demonstrates a reduction in oxidative stress in encapsulated chondrocytes in vitro and protects these cells from H_2_O_2_-induced phenotypic changes. In vitro cartilage construction experiments and in vivo subcutaneous implantation experiments reveal that the bioink facilitates the robust generation of ECM, leading to improved cartilage lumen formation and enhanced cartilage tissue regeneration. These results highlight the potential application of RTMA-1 bioink in cartilage tissue engineering, particularly in environments with oxidative stimuli, through 3D bioprinting.

## Figures and Tables

**Table 1:** Parameters for 3D printing.

Thickness (mm)	Section Numbers (100 μm/Layer)	Exposure Time per Layer (s)	Exposure Intensity (mW/cm^2^)
1	10	8	22 of the first layer, 14 of the rest

**Table 2:** The sequences of primers used for RT-qPCR.

The Full Name of Each RNA	Abbreviation	5′-3′	Primer Sequences
Glyceraldehyde-3-phosphate	*GAPDH*	Forward	TTGTCGCCATCAATGATCCAT
		Reverse	GATGACCAGCTTCCCGTTCTC
SRY-related HMG box 9	*SOX9*	Forward	GCGTCAACGGCTCCAGCAAGA
		Reverse	GCGTTGTGCAGGTGCGGGTAC
Aggrecan	*AGG*	Forward	GCTGCTACGGAGACAAGGATG
		Reverse	CGTTGCGTAAAAGACCTCACC
Type II Collagen	*COL II*	Forward	GAGAGCCTGGGACCCCTGGAA
		Reverse	CGCCTCCAGCCTTCTCGTCAA
Type I Collagen	*COL I*	Forward	CTAGCCACCTGCCAGTCTTTA
		Reverse	GGACCATCATCACCATCTCTG
Matrix metalloproteinase-1	*MMP1*	Forward	TTCCAAAGCAGAGAGGCAATG
		Reverse	CACCTGGGTTGCTTCATCATC
Matrix metalloproteinase-3	*MMP3*	Forward	GTGATACGCAAGCCCAGGTGT
		Reverse	CTCTTGGCAGATCCGGTGTGT
Matrix metalloproteinase-13	*MMP13*	Forward	GTCTTCTGGCTCACGCTTTTC
		Reverse	GGCAGCAACGAGAAACAAGTT

## Data Availability

Source data are available from the corresponding author upon reasonable request.
